# Validation of a high-risk versus low-risk referral model in suspected Acute Coronary Syndrome

**DOI:** 10.1186/1757-7241-20-S2-P51

**Published:** 2012-04-16

**Authors:** Christian Backer Mogensen, Maja Christiansen, Jess Bjerre Jørgensen, Peter Bisgaard Stæhr

**Affiliations:** 1 Emergency Department, Kolding Sygehus, Denmark

## Background

In future Denmark the majority of acute admissions passes through Emergency and Acute Admission Departments (EAAD). For patients with Acute Coronary Syndrome (ACS) considerable improvements have been achieved through the Cardiac Care Units (CCU). Among patients with acute chest pain only a minority have an ACS. This raises the question if it is possible to separate patients with a high-risk of ACS directly to a CCU, and a low-risk patients to EAAD. The aim of this study was to describe how such a risk stratification would perform.

## Methods

Retrospective cohort study of patients with suspicion of ACS. If the 1) ECG was normal (apart from atrial fibrillation), no persisting chest pain, no history of IHD, heart failure or an ICD, the patient was considered low-risk and admitted to the EAAD, otherwise to the CCU.

## Results

495 patients were admitted, 51 % low risk patients to the EAAD and 49 % with high risk to the CCU. 17 % had a verified ACS. Among ”low risk” patients 10 % and “high risk” 25 % had an ACS (p < 0.0001). In multivariate analysis high risk group, male gender and age above 60 years had a higher risk of ACS. Other combinations of risk factors increased the sensitivity at the expense of predictive value and accuracy.

## Conclusion

We believe the model can be useful for future development of referral strategies in acute hospitals in Denmark.

